# Disabling of lymphocyte immune response by Ebola virus

**DOI:** 10.1371/journal.ppat.1006932

**Published:** 2018-04-12

**Authors:** Patrick Younan, Mathieu Iampietro, Alexander Bukreyev

**Affiliations:** 1 Department of Pathology, University of Texas Medical Branch at Galveston, Galveston, Texas, United States of America; 2 Galveston National Laboratory, University of Texas Medical Branch at Galveston, Galveston, Texas, United States of America; 3 Department Microbiology & Immunology, University of Texas Medical Branch at Galveston, Galveston, Texas, United States of America; University of Kentucky, UNITED STATES

## Introduction

The lethality of the Ebola virus (EBOV) and its risk for public health came to the forefront during the 2013–2016 epidemic in West Africa. By the end of the epidemic, 28,616 suspected, probable, and confirmed cases of EBOV infection, including 11,310 fatalities, had been recorded [1]. For the first time, EBOV reached urban populations and demonstrated its full potential to spread rapidly and cause havoc before an effective containment response could be initiated. In this regard, EBOV can no longer be characterized as a virus that causes small sporadic outbreaks whose lethality limits the potential for larger epidemics. Furthermore, the continued growth of populations and urbanization within endemic regions may result in increased rates of EBOV outbreaks. Although several advancements have led to a basic understanding of the underlying mechanisms associated with the highly pathogenic nature of Ebola Virus Disease (EVD), a specific treatment for EBOV infection has yet to be approved.

## What is the association of lymphopenia with EVD outcome?

One of the identified links between survival and fatalities following EBOV infection is T-cell death resulting in their reduction in the peripheral blood. This condition, which is clinically known as lymphopenia, significantly impairs the body’s ability to fight infection, as T cells are critical to the development of a virus-specific, adaptive immune response. Lymphopenia and its occurrence in fatalities have been observed in mouse and nonhuman primate models of EVD in addition to humans with EVD [2–6]. Unlike HIV infection, in which CD4^+^ T cells are directly infected, both CD4^+^ and CD8^+^ T cells remain refractory to direct infection by EBOV; however, the absolute number of T lymphocytes in peripheral blood perceptibly drops, particularly in patients who succumb to EVD. Patients who survived EBOV infection are able to maintain T lymphocytes throughout the course of infection [7]. Below, we summarize the previously established mechanisms contributing to the onset of lymphopenia and highlight recent findings that further demonstrate the multifaceted causes that either directly or indirectly leads to T-cell depletion during EBOV infection.

## What are the indirect mechanisms of lymphopenia associated with EBOV infection?

### “Cytokine storm” and receptor-mediated cell death

A hallmark of EBOV infection is an overexasperated systemic release of inflammatory mediators collectively known as a “cytokine storm.” This burst in inflammatory mediators, which include the release of pro- and anti-inflammatory cytokines, chemokines, free radicals, and coagulation factors, is associated with continuously perpetuated signaling events [8]. The inability of the immune system to rein in this feedforward loop results in significant damage to tissue and organs [9]. Such an environment is toxic to T cells, and prolonged exposure results in bystander-mediated cellular death. In particular, Tumor necrosis factor α (TNFα) has been associated with the induction of both apoptotic and necrotic cell death [10].

A significant increase in TNF-related apoptosis-inducing ligand (TRAIL) expression has also been observed in human monocytes and macrophages during infection with EBOV *in vitro* [11], which can contribute to T-cell apoptosis. Lastly, reactive oxygen species (ROS) levels, which are highly toxic to lymphocytes [12], were significantly higher in human fatal cases in comparison to patients who survived EVD. A recent study demonstrated that survivors of the EBOV epidemic in West Africa exhibited significantly reduced levels of the overall inflammatory response compared to non-survivors [13]; hence, the key to improving the outcome following EVD may require timely administration of global immunosuppressive agents. In support of this prediction, our recent study demonstrated that administration of Eritoran, a Toll-Like Receptor 4 (TLR4) antagonist, significantly reduced the global inflammatory response and modified, but did not necessarily reduce the innate and adaptive immune responses, leading to increased survival in murine models of EVD and the disease caused by the closely related Marburg virus [14]. The role of EBOV-mediated interferon (IFN) antagonism in pathogenesis of EVD is well established [15]. Depletion studies in nonhuman primates demonstrated the involvement of T cells, B cells, and antibodies in vaccine-mediated protection against EBOV [16, 17], indirectly indicating their role in the natural protection against EBOV. Therefore, successful treatment of EBOV infections requires suppression of the pro-inflammatory component of the immune response without significantly sacrificing the quality and magnitude of both the innate and adaptive immune responses.

### Aberrant transmission of survival signals

**Fig 1:**
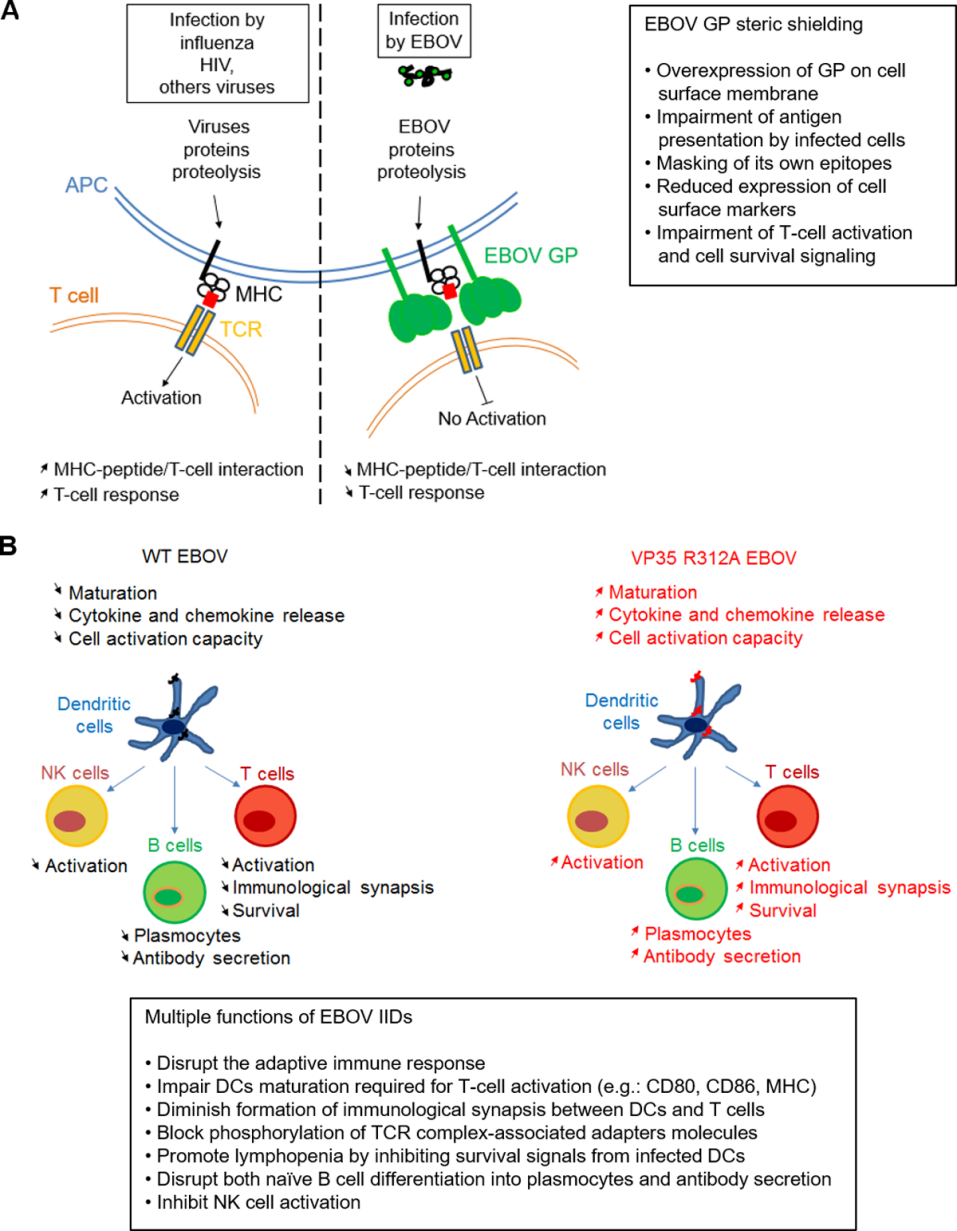
(A) Role of Evola virus (EBOV) glycoprotein (GP) steric shielding in impairment of T-cell response. Comparison between a typical viral infection and EBOV infection and the role of EBOV GP overexpression on cell surface membrane. (B) Role of EBOV VP35 interferon-inhibiting domain (IID) in suppression and dysregulation of cell-mediated response. Effects of wild-type (wt) EBOV (left) and the mutated virus with VP35 IID disabled by amino acid substitution R312A on dendritic cells (DCs), natural killer (NK) cells, B cells, and T cells. Summarized from references [20, 23, 24]. APC, antigen-presenting cells; CD-80, -86, cluster of differentiation; VP35, viral protein 35.

Recent reports have indicated the EBOV infection results in a substantial immune activation [13, 18]. Under more “normal” circumstances, such as infection with influenza virus, a significant contraction and depletion of activated cells is typically observed, with only a modest percentage of activated cells transitioning into effector memory T cells [19]. Although yet to be demonstrated *in vivo*, it is likely that the deficient T-cell response and lymphopenia in EBOV-infected patients or animal models of EVD are caused by a partial depletion of antigen-presenting cells (APCs) and/or deficient signaling events needed to initiate and maintain the transition into memory cells. This hypothesis is supported by previous findings demonstrating that the viral envelope glycoprotein (GP) masks its own epitopes as well as cellular surface proteins such as β1 integrin and MHC-I through steric shielding following GP expression on the plasma membrane of infected APCs [20] (Fig 1A). Furthermore, recent findings have demonstrated that EBOV-infected dendritic cells (DCs) have an impaired ability to activate T cells and exhibit a limited capacity to transmit survival signals to T lymphocytes [21, 22] (Fig 1B). Indeed, mechanistic studies demonstrated that biological effects of IFN-inhibiting domains (IIDs) located in EBOV proteins VP35 and VP24 lead to inhibition of T cell receptor (TCR) signal transduction following contact between EBOV-infected DCs and T cells, as disruption of IIDs by point mutations restored the transduction. Specifically, the VP35 and VP24 IIDs were found to cause deficient maturation of DCs [23], which, upon contact with T cells, did not induce phosphorylation of TCR complex–associated adapter molecules ZAP70, PLCγ1, and SLP76, resulting in impaired downstream signaling [24]. The IIDs were also found to inhibit differentiation and activation of human B cells contacting with EBOV-infected APCs. In particular, the rate of B cell differentiation from naïve to plasma cells was significantly increased in mixed peripheral blood mononuclear cell (PBMC) cultures infected with EBOV that was carrying mutated VP35 IID as compared to wild-type (WT) EBOV [24]. Surprisingly, the effect extended beyond T and B lymphocytes as the mutation also had a profound effect on human natural killer (NK) cell activation [24]. This can possibly be explained by some kind of interaction of NK cells with infected DCs, as antigen-specific memory-NK cells were recently identified in rhesus macaques [25]. While disabling of VP35 IID leads to EBOV attenuation *in vivo* [26], the available data do not distinguish between the roles of IFN antagonism from the IID-mediated suppression of cell-mediated response in this attenuation. These *in vitro* findings provide initial evidence demonstrating the role of EBOV IIDs in the deficient cell-mediated response, although further studies are required to demonstrate the relative importance of IID-mediated suppression of cell-mediated response for attenuation *in vivo*.

## Do EBOV virions directly interact with T lymphocytes?

**Fig 2:**
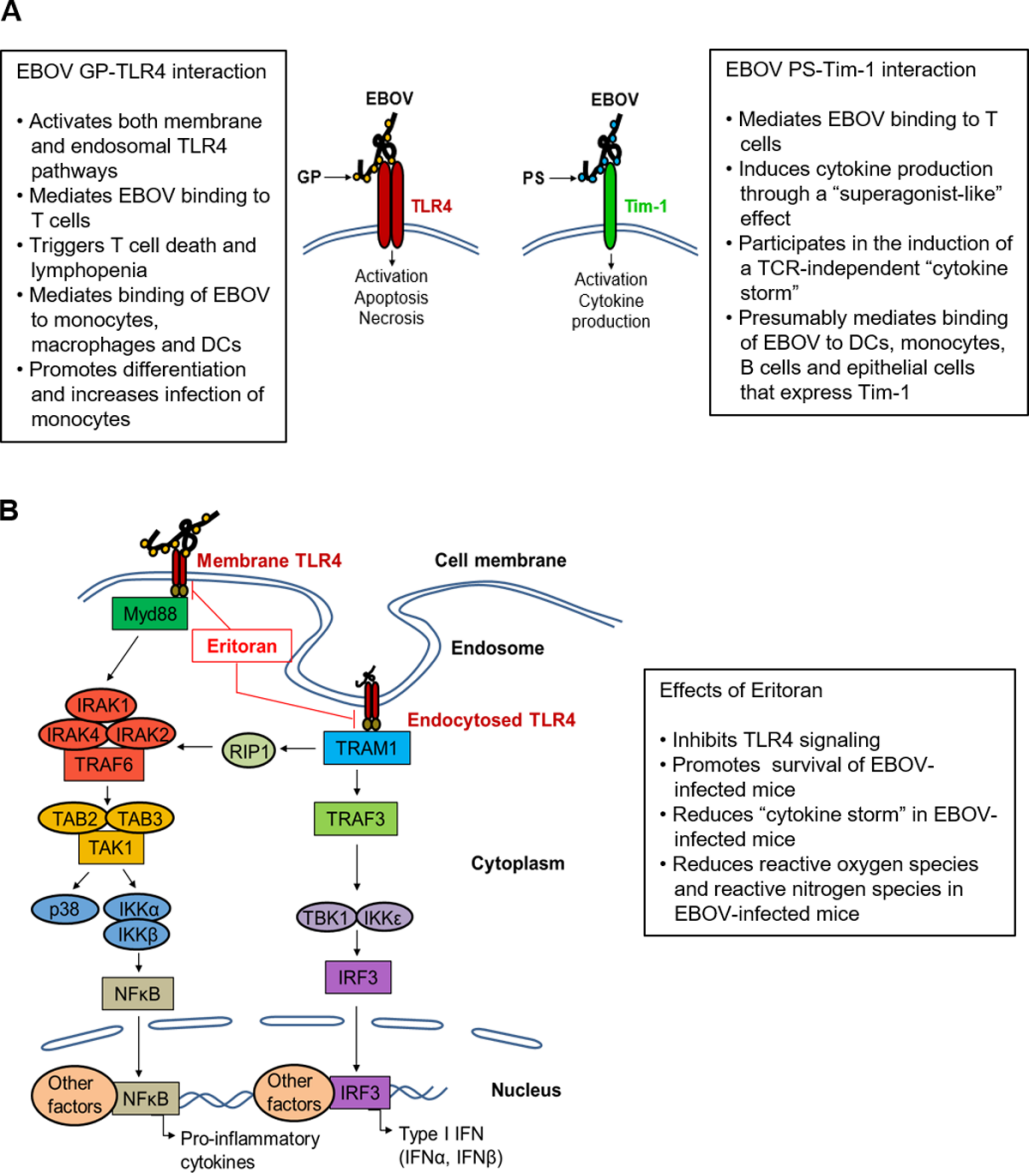
Mechanisms of Ebola virus (EBOV) interaction with T cells. (A) Role of Toll-Like Receptor 4 (TLR-4) and T-cell immunoglobulin-like protein 1 (Tim-1) in binding of EBOV to T cells. (B) Induction of TLR-4 signaling by EBOV and effects of Eritoran. Summarized from references [17, 27, 28]. PS, phosphatidylserine; TCR, T cell receptor; GP, glycoprotein; DC, dendritic cell; Myd88, myeloid differentiation primary receptor response 88; IRAK-1, -2, -4, interleukin-1 receptor-associated kinase 2, 3, 4; TRAF-3, -6, TNF Receptor Associated Factor 3, 6; TAB-2, -3, TGF-beta-activated kinase 1/MAP3K7 Binding Protein 2; TAK1, transforming growth factor beta activated kinase 1 (MAP3K7); p38, mitogen-activated protein kinase; IKKα, -β, -ε, inhibitor of nuclear factor kappa kinase; NFκB, nuclear factor kappa-light-chain-enhancer of activated B cells; RIP1, receptor-interacting protein 1; TRAM1, translocation associated membrane protein 1; TBK1, TANK binding kinase 1; IRF3, interferon regulatory factor 3; IFNα, -β, interferon α, -β.

Although the addition of EBOV to isolated T lymphocytes does not result in a productive infection, EBOV virions have recently been shown to directly bind to and activate CD4^+^ T cells [27, 28]. Binding and activation of CD4^+^ T cells were shown to occur through at least two identified interactions: between GP with TLR4 [27], which is consistent with previous studies showing interaction of envelope proteins of a number of viruses with pattern recognition receptors [29], and between the viral membrane–associated phosphatidylserine (PtdSer) and T-cell immunoglobulin-like protein 1 (Tim-1) [28] (Fig 2A and 2B). In both cases, the direct binding of EBOV virions to CD4^+^ T cells was shown to result in a burst in cytokine production and ultimately to lead to increased cell death in *in vitro* experiments. The findings suggest that EBOV induces activation of T cells regardless of antigenic specificity of their receptors, which is consistent with recent findings in EBOV-infected patients [13].

As a multitude of enveloped viruses have been shown to incorporate PtdSer into their viral membranes as a means of apoptotic mimicry [30], which serves to enhance infectivity, broaden tropism, and promote immune evasion, it is likely that binding of virion-associated PtdSer to Tim-1 may contribute to the pathogenesis of a broad range of viruses. This prospect is supported by findings showing that targeting PtdSer in guinea pigs infected with Lassa virus protects animals from death [31]. Similarly, infection of Tim-1-knockout mice with EBOV results in a significant survival not observed in WT control mice [28]. Further investigation into the mechanism of PtdSer incorporation into budding EBOV virions and the feasibility of targeting PtdSer–Tim-1 interactions may result in development of treatments.

All current EBOV vaccines use GP as the sole antigen, which induces protective immune response. Immunization with the vaccine based on the vesicular stomatitis virus–(VSV) based vector during the EBOV epidemic in Guinea in 2015 demonstrated 100% protective efficacy [32], which is a great success, and a recent study demonstrated an acceptable safety profile of the vaccine [33]. However, a well-controlled clinical study of the VSV-vectored vaccine in Europe demonstrated adverse effects, including arthritis, vasculitis, and dermatitis, which were not observed in the control group of vaccine recipients that received an empty VSV vector, resulting in halting of the trials involving the doses expected to be minimally sufficient for protection (1 × 10^7^ and 5 × 10^7^ PFU) [34]. These effects are likely to be related to pro-inflammatory and immunomodulating effects of GP discussed above in the “Cytokine storm” and receptor-mediated cell death section.

## Conclusions

It has become clear that pronounced lymphopenia is intimately linked with fatalities caused by EBOV as well as a broad range of pathogens. Notably, this occurrence is a common characteristic of some of the most lethal human diseases: viral hemorrhagic fevers, infections with highly pathogenic influenza viruses, and bacterial sepsis [35]. Furthermore, it is likely that several of the mechanisms that lead to lymphopenia during these conditions are also at play during the acute phase of chronic infections, causing transient lymphopenia as a result of bystander-mediated cell death (e.g., acute phase of HIV infection). Hence, continually expanding our knowledge of the mechanisms by which viruses cause lymphopenia in the absence of direct infection of lymphocytes may greatly aid in the development of future broad-acting therapeutics that limit or prevent premature lymphocyte death. Such therapies would putatively increase the chances of mounting an antigen-specific adaptive immune response, which would lead to better patient outcome.
